# EWS-FLI1 confers exquisite sensitivity to NAMPT inhibition in Ewing sarcoma cells

**DOI:** 10.18632/oncotarget.14976

**Published:** 2017-02-01

**Authors:** Cornelia N. Mutz, Raphaela Schwentner, Dave N.T. Aryee, Eric D.J. Bouchard, Edgard M. Mejia, Grant M. Hatch, Maximilian O. Kauer, Anna M. Katschnig, Jozef Ban, Antje Garten, Javier Alonso, Versha Banerji, Heinrich Kovar

**Affiliations:** ^1^ Children's Cancer Research Institute Vienna, St. Anna Kinderkrebsforschung, Vienna, Austria; ^2^ Department of Biochemistry and Medical Genetics, University of Manitoba, Research Institute in Oncology and Hematology (RIOH), CancerCare Manitoba, Winnipeg, Canada; ^3^ Department of Pharmacology and Therapeutics, Faculty of Health Sciences, University of Manitoba, Winnipeg, Canada; ^4^ Department of Biochemistry and Medical Genetics, Center for Research and Treatment of Atherosclerosis, University of Manitoba, DREAM Children's Hospital Research Institute of Manitoba, Winnipeg, Canada; ^5^ Department of Pediatrics, Medical University Vienna, Vienna, Austria; ^6^ Center for Pediatric Research Leipzig, Hospital for Children and Adolescents, University of Leipzig, Leipzig, Germany; ^7^ Unidad de Tumores Sólidos Infantiles, Instituto de Investigación de Enfermedades Raras, ISCIII, Ctra, Madrid, Spain

**Keywords:** EWS-FLI1, Ewing sarcoma, NAMPT, NAD, FK866

## Abstract

Ewing sarcoma (EwS) is the second most common bone cancer in children and adolescents with a high metastatic potential. EwS development is driven by a specific chromosomal translocation resulting in the generation of a chimeric EWS-ETS transcription factor, most frequently EWS-FLI1.

Nicotinamide adenine dinucleotide (NAD) is a key metabolite of energy metabolism involved in cellular redox reactions, DNA repair, and in the maintenance of genomic stability. This study describes targeting nicotinamide phosphoribosyltransferase (NAMPT), the rate-limiting enzyme of NAD synthesis, by FK866 in EwS cells. Here we report that blocking NAMPT leads to exhaustive NAD depletion in EwS cells, followed by a metabolic collapse and cell death. Using conditional EWS-FLI1 knockdown by doxycycline-inducible shRNA revealed that EWS-FLI1 depletion significantly reduces the sensitivity of EwS cells to NAMPT inhibition. Consistent with this finding, a comparison of 7 EwS cell lines of different genotypes with 5 Non-EwS cell lines and mesenchymal stem cells revealed significantly higher FK866 sensitivity of EWS-ETS positive EwS cells, with IC_50_ values mostly below 1nM.

Taken together, our data reveal evidence of an important role of the NAMPT-mediated NAD salvage pathway in the energy homeostasis of EwS cells and suggest NAMPT inhibition as a potential new treatment approach for Ewing sarcoma.

## INTRODUCTION

Reprogramming cellular metabolism is a key mechanism of tumorigenesis and has recently been assigned a hallmark of cancer [1]. One approach to taking advantage of the metabolic dependencies of a cancer is to deplete the tumor cells from nicotinamide adenine dinucleotide (NAD) [2]. NAD is an important co-factor of cellular redox reactions, a substrate for a variety of NAD-dependent enzymes and regulatory proteins, and acts as a key regulator of energy metabolism and signal transduction [3, 4]. Cancer cells have a particularly high demand for NAD due to their fast proliferation [5] and are more dependent on salvage pathways required for fast NAD regeneration and survival than normal cells [6]. In mammals, NAD can either be synthesized *de novo* from the essential amino acid tryptophan (TRP) or, in NAD salvage pathways, from precursors such as nicotinamide (NAM), nicotinamide mononucleotide (NMN), nicotinic acid (NA), or nicotinamide riboside (NR) [4]. The rate-limiting enzyme of mammalian NAD biosynthesis starting from NAM is nicotinamide phosphoribosyltransferase (NAMPT), a cytosolic enzyme transferring a phosphoribosyl group from 5-phosphoribosyl-1-pyrophosphate (PRPP) to NAM forming NMN [7, 8]. Subsequently, NAD is synthesized from NMN and adenosine triphosphate (ATP) via the NMN adenylyltransferase [9]. NAMPT is crucial for replenishment of the intracellular NAD pool, and in several cancer types - including prostate, gastric, breast, and ovarian cancer, gliomas, leukemia, lymphoma, and myeloma - NAMPT was found to be overexpressed [10] and associated with disease progression [11]. Reducing the availability of NAD in cancer cells interferes with tumor progression on one hand by impairing cellular energy metabolism and on the other hand by limiting the activity of NAD-dependent enzymes such as sirtuins and poly-(ADP-ribose) polymerases (PARPs) [3]. PARP1 is a post-translational modifier operating as a DNA repair enzyme on DNA strand breaks, which recruits other proteins via the formation and attachment of mono- or polymers of ADP-ribose. It maintains genome stability and regulates transcription by modulating chromatin structure [12]. Likewise, sirtuins act as post-translational modifiers by de-acetylating histone and non-histone proteins in response to stress [13]. Thus, NAD levels not only impact on energy metabolism but also on the cell's propensity to sense and respond to various types of cellular stress.

PARP1 as well as the sirtuin SIRT1 are highly expressed in Ewing sarcoma (EwS), the second most common primary malignant bone tumor in children and adolescents, downstream of the driver oncogene EWS-FLI1 [14–16]. This aberrant transcription factor results from the translocation t(11;22)(q24;q12) fusing the Ewing sarcoma breakpoint region 1 (*EWSR1*) gene with Friend leukemia virus integration site 1 (*FLI1*) in 85% of EwS [17, 18]. In the remaining cases, alternative gene fusions between *EWSR1* and one of four related ETS transcription factor genes – *ERG*, *ETV1*, *ETV4*, or *FEV* – are found [19].

EWS-FLI1 expression was described as sensitizer to PARP inhibition [20], and targeting PARP1 by immobilizing it on DNA double strand breaks has been proposed as a treatment strategy for EwS [21]. However, single agent clinical trials have not been successful so far and combination chemotherapy with PARP inhibitors and DNA damaging drugs is currently under investigation [21–23].

Because of the high expression of NAD-consuming enzymes in EwS cells, we tested whether they might be specifically sensitive to NAMPT inhibition. Intriguingly, we found potent antitumor activity of the NAMPT inhibitor FK866 (*N*-[4-(1-benzoyl-4-piperidinyl)butyl]-3-(3-pyridinyl)-2*E*-propenamide), also known as APO866 or WK175, in the low nanomolar range. FK866 was previously identified by screening for interference with growth regulation in cancer cells [24]. It was described to mediate delayed cell death via apoptosis, oncosis, or autophagy [25–28]. The compound gradually exhausts cellular NAD levels via competing with NAM for binding to the NAMPT dimer interface, thereby specifically inhibiting NAMPT enzymatic activity [29]. FK866 has been evaluated as anticancer agent in several hematologic malignancies [30–32], solid tumors [33] and cancer cell lines [26, 34, 35]. Thus, disrupting cancer cell metabolism via reduction of NAD, one of the key metabolites of cellular redox reactions and ATP-synthesizing mitochondrial electron transport chain reactions [3], might become an attractive target in cancer therapy.

In this study, we interrogated the effects of pharmacological inhibition of NAMPT by FK866 on cellular energy content, cell death, mitochondrial dysfunction, and glycolysis in EwS cells. We demonstrate that expression of EWS-FLI1 increases the susceptibility to FK866-induced NAD depletion, apoptosis, and glycolytic stress. These findings suggest that inhibiting NAMPT-mediated NAD salvage might represent a novel approach to attenuate EwS cell proliferation by blocking the engine of the disease.

## RESULTS

### NAMPT plays a key role in maintaining NAD levels in EwS cells

**Figure 1:**
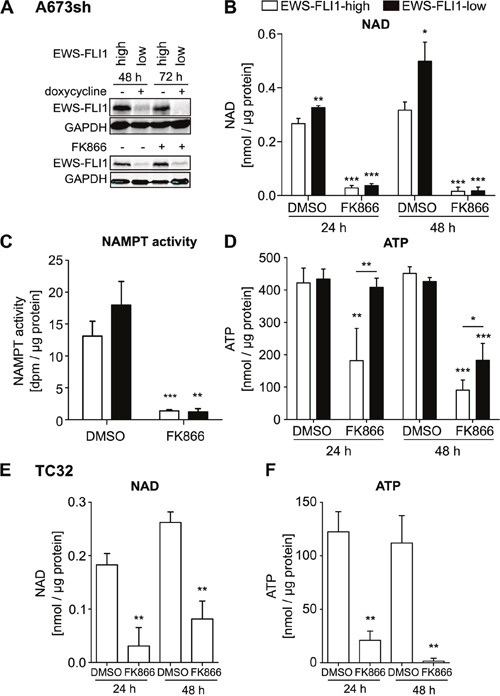
NAMPT inhibition by FK866 reduces NAD and ATP in EwS cells A673sh and TC32 **A**. Addition of doxycycline in A673sh cells allows for switching from high (EWS-FLI1-high, EFH) to low (EWS-FLI1-low, EFL) EWS-FLI1 expression levels. A673sh were doxycycline (dox) treated or not and protein lysates were obtained and immunoblotted after 48 h and 72 h induction of the shRNA against EWS-FLI1 (upper blot). 24 h after dox addition, cells were treated with 2 nM FK866 or solvent (DMSO) for 24 or 48 h (lower blot). Western blots shown are representative pictures demonstrating efficacy of doxycycline-induced EWS-FLI1 knockdown, and illustrate that FK866 does not interfere with EWS-FLI1 expression. **B**. NAD content in EFH and EFL (48 h / 72 h dox) A673sh cells after 24 h and 48 h of 2 nM FK866 treatment. **C**. NAMPT activity was determined from cell lysates of EFH and EFL (48 h dox) after application of 5 nM FK866 for 24 hours. **D**. ATP content in EFH and EFL (48 h / 72 h dox) A673sh cells after 24 h and 48 h of 2 nM FK866 treatment. **E**. NAD and **F**. ATP abundance in TC32 EwS cells before and after addition of 2 nM FK866 or DMSO for 24 h and 48 h. Results were normalized to total protein amount in each sample and cells were cultivated in medium + 10% FCS. DMSO was used as vehicle/control. Data illustrate the means ± SD of a minimum of three independent experiments (*P<0.05, **P<0.01, ***P<0.001).

To obtain a profile of cellular energy content, we investigated NAD and ATP levels in A673sh cells, which conditionally express shRNA to EWS-FLI1 upon doxycycline treatment, thus resulting in EWS-FLI1-high (EFH) (no doxycycline) and EWS-FLI1-low (EFL) (48 / 72 h doxycycline treatment) protein expressing cells (Figure 1A). EFH cells contained significantly less NAD than EFL cells (Figure 1B: 48 h: 0.267±0.019 in EFH versus 0.326±0.008 nmol/μg protein in EFL cells; 72 h: 0.317±0.03 in EFH versus 0.499±0.071 nmol/μg protein in EFL cells). Furthermore, we found that NAMPT inhibition by FK866 acted specifically via reducing NAMPT activity in A673sh cells and had no effect on EWS-FLI1 protein levels (Figure 1A & 1C). Under both conditions, EFH and EFL, NAMPT activity was significantly and similarly reduced after treatment with FK866 as indicated by a dramatic loss of NAD in A673sh cells at 24 h and almost no detectable NAD after 48 h inhibitor treatment (Figure 1B). Following NAD depletion, ATP levels declined in a delayed manner (Figure 1D) which may be the consequence of cellular energy stress and ensuing cell death in response to FK866 as was previously reported for hepatocarcinoma cells [35]. A similar reduction in NAD and ATP levels was observed in TC32 EwS cells, which independently validates our results in A673sh (Figure 1E-1F). In TC32, there was a slight tendency that NAD levels are higher after 48 hours than after 24 hours of FK866 treatment. However, this difference did not achieve statistical significance (p = 0.14). Interestingly, under short (24 h) and long term (48 h) FK866 treatment conditions, EFL cells sustained their ATP levels much better than EFH cells (Figure 1D). Maintenance of higher ATP levels in FK866 treated EFL versus EFH cells suggests diminished vulnerability to NAMPT inhibition possibly due to EWS-FLI1 knockdown-induced cell cycle arrest [36].

### The effect of FK866 is rescued by NMN or NA supplementation

**Figure 2:**
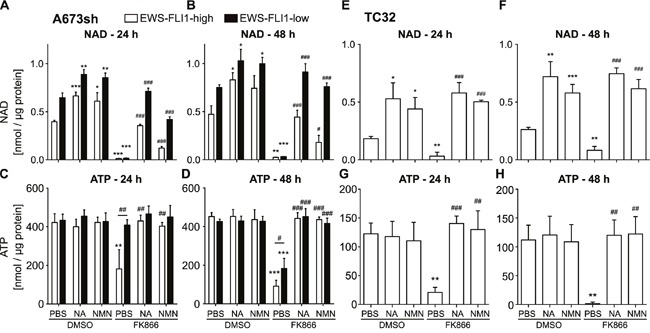
Supplementation with metabolites of the NAD salvage pathway can restore NAD and ATP levels after NAMPT inhibition **A-B**. NAD and **C-D**. ATP levels in A673sh cells which were treated with 25 μM NA or 500 μM NMN (dissolved in PBS) 6 h before and throughout the addition of 2 nM FK866 for 24 h (A & C) and 48 h (B & D) in EFH and EFL (48 / 72 h dox) conditions. Cells were cultivated in serum-containing medium and DMSO-vehicle served as control for FK866 treatment. **E-F**. NAD and **G-H**. ATP content in TC32 cells with supplementation of NA and NMN (as described above) and treatment with 2 nM FK866 for 24 h or 48 h. Results were normalized to total protein amount and data represent the means ± SD of a minimum of three independent experiments (*P<0.05, **P<0.01, ***P<0.001 compared to DMSO-PBS; #P<0.05, ##P<0.01, ###P<0.001 compared to FK866-PBS).

Because NAD is rapidly consumed in cells [37], fast regeneration needs to be accomplished via distinct NAD salvage pathways, starting either from NAM via NMN, NA, or NR [4]. We tested whether supplementation with NMN or NA could rescue the effects of NAMPT inhibition. NAMPT produces NMN from NAM, and NA is initially converted into nicotinic acid mononucleotide (NaMN) by nicotinic acid phosphoribosyltransferase (NAPRT) in order to fuel NAD pools [38]. The effect of FK866 on NAD abundance in A673sh cells could be largely restored by adding NA or NMN (Figure 2A-2B). However, only NA accomplished a full rescue up to normal levels (DMSO-PBS) for 24 and 48 h FK866 treatment in EFH cells. In contrast, addition of NMN led to only a 30% and 37% recovery of NAD levels at 24 h (Figure 2A: 0.396±0.014 nmol/μg protein in DMSO-PBS vs 0.121±0.017 nmol/μg protein in FK866-NMN) and 48 h (Figure 2B: 0.472±0.089 nmol/μg protein in DSMO-PBS vs 0.179±0.074 nmol/μg protein in FK866-NMN), respectively. Interestingly, cells with low EWS-FLI1 expression could efficiently compensate FK866-induced NAD depletion with supplementation of either metabolite (NMN or NA) under short (24 h, Figure 2A) and long (48 h, Figure 2B) NAMPT inhibition conditions. The only exception for full NAD replenishment was observed for short term NMN supplementation, which allowed for only a 65% recovery in NAD levels in EFL cells (0.646±0.05 nmol/μg protein in DMSO-PBS versus 0.420±0.028 nmol/μg protein in FK866-NMN). ATP levels, however, reached normal levels upon rescue with both NMN and NA in EFH and EFL EwS cells (Figure 2C-2D). In TC32 cells, NA and NMN equally rescued NAD and ATP abundance after NAMPT inhibition (Figure 2E-2H). In comparison to A673sh cells, TC32 cells have lower NAD (Figure 2A-2B & 2E-2F) and ATP pools (Figure 2G-2H) in general. In the absence of FK866, both NA and NMN commonly increased NAD contents in both cell lines (Figure 2A-2B & 2E-2F), suggesting immediate availability of NAD regeneration via salvage pathways in EwS cells.

### NAMPT inhibition leads to decrease of mitochondrial respiration and suppression of glycolysis

**Figure 3:**
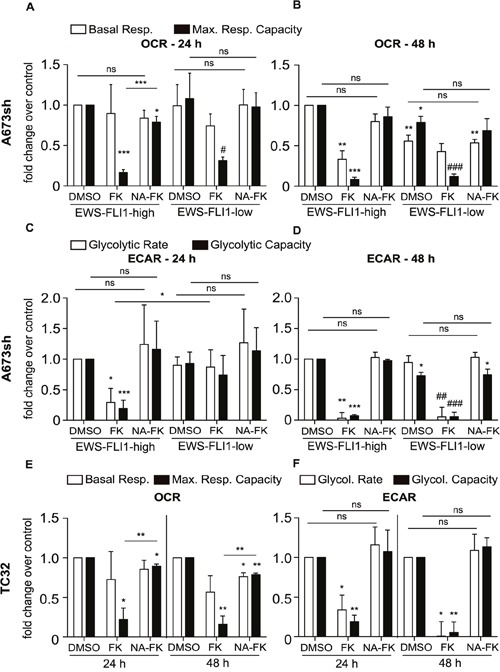
Bioenergetic profiles of EFH and EFL A673sh and of TC32 cells O_2_ consumption rates (OCR) and extracellular acidification rates (ECAR) were measured in real time and after injection of mitochondrial / glycolytic inhibitors as described in “Materials and Methods”. **A-D**. 1×10^4^ A673sh cells were seeded and treated with doxycycline for 17 h the next day. Cells were pre-treated with 25 μM NA (NA-FK) on the following day for 6 h before and throughout the addition of 2 nM FK866 (FK) for a period of 24 h (A & C) or 48 h (B & D). The results were normalized to total protein after measurement. Basal respiration (BR) and maximal respiratory capacity (MRC) as well as glycolytic rate (GR) and glycolytic capacity (GC) data were normalized to DMSO-EFH and displayed as fold change over control. **E-F**. 9×10^3^ TC32 cells were seeded and pre-treated with 25 μM NA (NA-FK) on the following day 6 h before and throughout the addition of 0.4 nM FK866 (FK) for a period of 24 h (E) or 48 h (F). The results were normalized to total protein after measurement. BR, MRC, GR, and GC results were normalized to the respective DMSO-vehicle control (24 h or 48 h) and displayed as fold change over control. Data are represented as the means ± SD from a minimum of three independent replicates (*P<0.05, **P<0.01, ***P<0.001 compared to DMSO-EFH; #P<0.05, ##P<0.01, ###P<0.001 compared to DMSO-EFL, ns: not significant).

To better understand how NAMPT inhibition leads to changes in EwS cell metabolism, we evaluated mitochondrial respiration and glycolysis in A673sh (EFH and EFL) and TC32 cells in the presence and absence of FK866 (24 h or 48 h) as well as with and without addition of NA. The Seahorse XF24 analyzer was used to determine oxygen consumption rates (OCR) as readout for mitochondrial respiration and extracellular acidification rates (ECAR) as a measure for glycolysis in confluent A673sh and TC32 cells (Figure 3). OCR, represented by basal respiration (BR) and maximal respiratory capacity (MRC), was significantly diminished after 48 h NAMPT inhibition in EFH cells (Figure 3B), whereas after 24 h treatment, mostly MRC was affected in EFH and EFL expressing cells (Figure 3A). BR could be maintained during short term NAMPT inhibition (Figure 3A), however, after 48 h drug treatment, BR was significantly lowered in EFH, but not in EFL cells (Figure 3B). Interestingly, we observed lowered BR and MRC in EFL control (DMSO) treated cells compared to EFH cells (Figure 3B), which might represent a doxycycline effect [39]. However, doxycycline treatment of parental A673 cells had no effect on ATP levels and cell viability (Supplementary Figure 1), and the relative effect of FK866 on OCR did not differ from EFH to EFL conditions. Thus, it is unlikely that doxycycline treatment had any influence on our results other than reducing EWS-FLI1 levels through shRNA mediated knockdown. For both treatment periods, supplementation with NA could fully or at least partially restore BR and MRC levels (Figure 3A-3B). The effect of NAMPT inhibition on OCR showed the same response in EFH and EFL cells for MRC, but not for BR, which was significantly decreased in EFL cells after 48 h treatment (Figure 3B). However, glycolytic stress tests in EFL compared to EFH cells revealed a delayed response in reduction of glycolytic rate (GR) and glycolytic capacity (GC) after 24 h NAMPT inhibition (Figure 3C). At a time when EFH-FK866 treated cells showed diminished GR and GC compared to EFH-DMSO control treated cells, EFL cells still maintained indistinguishably high GR and GC under FK866 treatment (Figure 3C). During 48 h NAMPT inhibition (Figure 3D), EWS-FLI1 silencing did not exert a rescue effect on glycolysis anymore and for both high and low EWS-FLI1 expression, FK866 application resulted in a loss of GR as well as GC (Figure 3D). As already confirmed for OCR measurements, also during the glycolytic stress test, addition of the NAD salvage fueling metabolite NA resulted in a full restoration of ECAR (Figure 3C-3D). NAMPT inhibition in TC32 cells led to prominently decreased MRC, but basal respiration was not much affected (Figure 3E). In concordance with measurements in A673sh cells, TC32 displayed glycolytic dysfunction after NAMPT inhibition, resulting in decreased GR and GC (Figure 3F). Again, NA addition counteracted the effect of FK866 (Figure 3E-3F). Hence, EwS cells exhibited mitochondrial and glycolytic dysfunction upon NAMPT inhibition, and glycolysis in EWS-FLI1 expressing cells was more susceptible to short term FK866 treatment than in EWS-FLI1 depleted cells.

### FK866-induced NAMPT inhibition blocks DNA synthesis and induces EwS cell death at sub-nanomolar concentrations dependent on EWS-FLI1 expression

**Figure 4:**
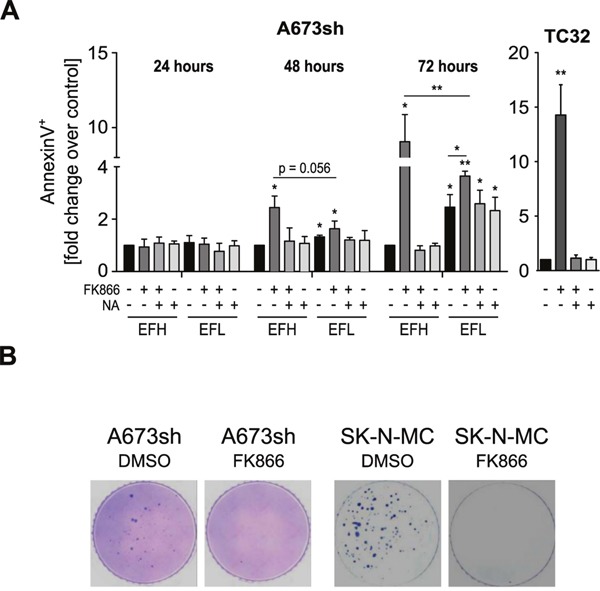
FK866-induced NAMPT inhibition causes EwS cell death and loss of clonogenic growth **A**. AnnexinV/DAPI staining of A673sh and TC32 cells treated with 5 nM FK866, FK866 + NA, or NA alone. Cells were pre-treated with 25 μM NA for 6 h before and throughout the addition of FK866, 24-72 h to A673sh and 72 h to TC32. The percentage of AnnexinV-positive/DAPI-negative cells from adherent and floating cells was determined, normalized to EFH-DMSO control (set to 1), and displayed as relative fold change. Cells were cultivated in serum-containing medium and DMSO was used as vehicle control. Data are shown as means ± SD from three independent experiments (*P<0.05, **P<0.01, ***P<0.001; ns: not significant; EFH: EWS-FLI1-high; EFL: EWS-FLI1-low). **B**. Clonogenicity of A673sh and SK-N-MC cells was investigated by soft agar colony formation assays. Cells were treated once with 2 nM of FK866 or DMSO (control) at time of seeding, fed with fresh medium every three days, and pictures were taken 21 days after seeding. A representative of three experiments is shown.

**Figure 5:**
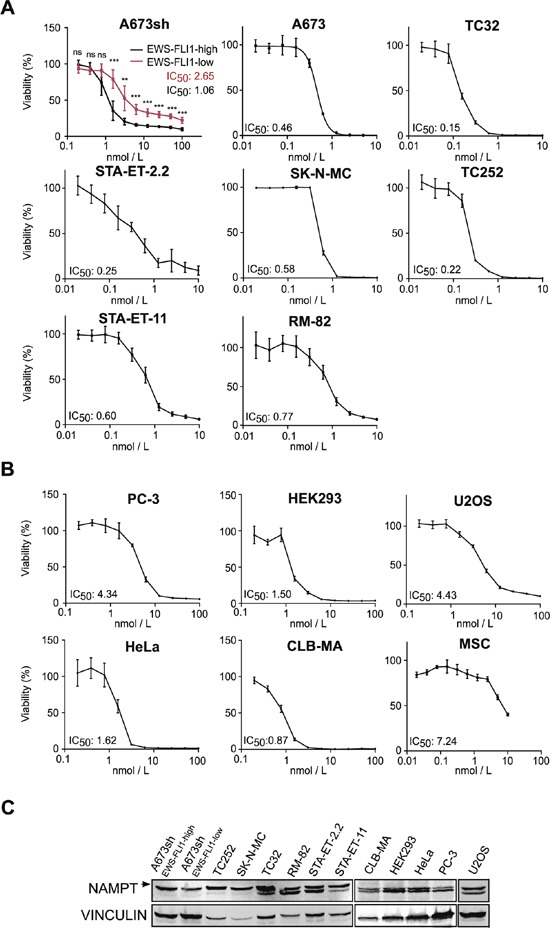
FK866 decreases cell viability in a sub-nanomolar range in EwS cells Dose-response curves to FK866 treatment and determination of IC_50_ values for **A**. A673sh cell clone at EFH and EFL conditions, A673 parental cell line and six additional EwS cell lines harboring different EWS-FLI1 and EWS-ERG fusion types (Supplementary Table 1) and **B**. six non-EwS cell types: PC-3 (prostate cancer), HEK293 (kidney), HeLA (cervical carcinoma), CLB-MA (neuroblastoma), U2OS (osteosarcoma), and MSCs. 4×10^3^ A673sh cells were seeded in each well of 96-well plates, treated or not with doxycycline the next day, and were compound treated for additional 72 h starting 24 h after doxycycline administration. 1-1.2×10^4^ cells of the other non-inducible cell lines were seeded in 96-well plates and treated with FK866 for 72 h the next day. Data represent the means ± SD from a minimum of three independent experiments (*P<0.05, **P<0.01, ***P<0.001). **C**. Immunoblot analysis for NAMPT expression (arrow) in different EwS and Non-EwS cell lines. Protein band for EWS-FLI-low band shows cells after 48 h doxycycline treatment. Vinculin was used as loading control.

NAMPT inhibition-induced NAD depletion results in broad metabolic defects and has been shown to reduce mitochondrial membrane potential which can subsequently lead to cell death [25, 28, 31, 40]. We demonstrate that also in EwS cells, FK866 treatment leads to mitochondrial dysfunction (Figure 3), DNA synthesis blockade (Supplementary Figure 2A), and cell death (Figure 4A and Supplementary Figure 2B). EdU incorporation assays in A673sh and SK-N-MC cells treated with 5 nM FK866 for 72 h completely abolished S-phase, indicating strong cell cycle inhibition (Supplementary Figure 2A). Double staining with AnnexinV and DAPI revealed cell death induction without loss of plasma membrane integrity consistent with apoptosis (Supplementary Figure 2B). However, in contrast to Etoposide, a classical apoptosis inducer in EwS, FK866 treatment did not result in PARP1 cleavage, excluding a caspase-driven cell death mechanism (Supplementary Figure 2C). In A673sh, we observed a significant increase of 2.4 ± 0.44 fold in AnnexinV-positive/DAPI-negative cells already after 48 h of FK866 treatment under EFH conditions versus only 1.63 ± 0.29 fold in EFL cells. This difference was further markedly increased after 72 h of NAMPT inhibition, when the AnnexinV-positive/DAPI-negative fraction increased to 9.1 ± 1.8 fold in EFH versus 3.6 ± 0.2 fold in EFL conditions (Figure 4A). NA supplementation (25 μM) reduced the proportion of dead cells to basal levels which, at 72 h, was slightly higher in EFL than in EFH cells. In TC32 cells, FK866 treatment (5 nM) for 72 h induced an increase in AnnexinV-positive/DAPI-negative cells of 14.3 ± 2.8 fold, which was fully rescued by the addition of NA. To validate these results, cell viability under monolayer growth conditions was assessed in response to increasing doses of FK866 in A673sh under EFH and EFL conditions and a total of seven EwS cell lines harboring different gene rearrangements, such as *EWS-FLI1* exon 7/6 fusion (A673, TC32, SK-N-MC, TC252), *EWS-FLI1* exon 9/4 fusion (STA-ET-2.2), and *EWS-ERG* fusion (STA-ET-11, RM-82), summarized in (Supplementary Table 1 and Figure 5A).

We compared these results to those obtained from the analysis of five Non-EwS cell lines, PC-3 (prostate cancer, *TMPRSS2-ERG* fusion), HEK293 (embryonic kidney), HeLa (cervical cancer), CLB-MA (neuroblastoma), and U2OS (osteosarcoma), and to the putative progenitor cell type of EwS, mesenchymal stem cells (MSCs) (Figure 5B). Intriguingly, despite comparable NAMPT protein levels (Figure 5C), the IC_50_ of the A673sh clone shifted from 1.06 in EFH to 2.65 nM in EFL conditions, as a consequence of significantly elevated viability values of EFL compared to EFH cells for each individual inhibitor concentration tested (Figure 5A). Generally, EwS cells showed a striking sensitivity to FK866 with IC_50_ values in the sub-nanomolar range (mean 0.51±0.31 nM), while IC_50_ values in the tested non-EwS cell lines were significantly higher (mean 2.55±1.52 nM) (p= 0.006). No correlation between NAMPT protein expression and FK866 sensitivity was observed (Figure 5C). FK866 toxicity not only affected EwS cells under monolayer but also under anchorage-independent growth conditions, a hallmark of oncogenic transformation [41]. A single drug administration at the time of seeding into soft-agar sufficed to fully suppress colony formation of A673sh and SK-N-MC (Figure 4B).

Together, these observations strongly suggest an EWS-ETS fusion-dependent exquisite susceptibility of EwS cells towards NAMPT inhibition.

## DISCUSSION

In the 1920s Otto Warburg and colleagues made the observation that tumor cells have a particularly high demand for glucose uptake in order to increase biomass [42]. Increased glycolysis in the presence of oxygen by fueling less pyruvate into oxidative phosphorylation and ATP production is termed “aerobic glycolysis” since then [43] and nowadays known as Warburg effect [42]. Aberrant cellular metabolism in cancer development has gained tremendous interest over the years and is discussed as a promising target in cancer treatment [1]. In support of this notion, targeting NAD biosynthesis has emerged as an innovative approach against tumor progression [2], and especially inhibiting the rate-limiting step of NAD generation via NAMPT has been extensively studied *in vitro* and *in vivo* [24, 27, 44]. NAD is extensively consumed during enhanced glycolysis and has an important function as a co-enzyme for mitochondrial electron transport chain reactions [45]. NAD is also a co-substrate for stress sensory protein modification pathways [46].

In this study, we have interrogated for the first time the metabolic consequences of NAMPT inhibition in EwS cells. We demonstrated that NAMPT activity is crucial for EwS cell survival, and that NAMPT inhibition by FK866 is synthetic lethal with EWS-FLI1 expression at sub-nanomolar inhibitor concentrations. Importantly, we showed efficient depletion of NAD by using low (0.4 – 5 nM) concentrations of FK866, followed by blockade of glycolytic capacity, mitochondrial dysfunction, ATP exhaustion and finally cell death. By using a doxycycline-inducible EWS-FLI1 knockdown approach, we found that NAMPT inhibition in the absence of the oncogene maintained functional glycolysis for a longer time period than in presence of EWS-FLI1. Glycolytic protection, low apoptosis, and an IC_50_ increase by more than 2-fold in EFL cells led us to the conclusion that EWS-FLI1 expressing cells are extremely sensitive to NAMPT inhibition which could be of therapeutic relevance. A comprehensive study of NAMPT inhibition in various tumor cell types *in vitro* and *in vivo* impressively demonstrated FK866-induced blockade of glycolysis, serine biosynthesis, tricarboxylic acid (TCA) cycle, and alterations in the pentose phosphate pathway [47]. Thus, it is likely that exhaustion of glycolysis also prohibits carbon flow to the TCA cycle in EwS cells resulting in ATP depletion and cell death. Cell viability assays were performed in cells with non-functional *p53* (A673sh, SK-N-MC, STA-ET-2.2, RM-82, PC-3, HEK293, HeLa) or wild-type *p53* (TC32, T252, STA-ET-11, CLB-MA, U2OS), and, in disagreement with a previous study of myeloid leukemias [48], we observed high response to NAMPT inhibition in EwS cells with disrupted *p53*, suggesting that FK866-induced cell death does not depend on functional p53. These observations are in line with results obtained for chronic lymphocytic leukemia [31] and hepatocarcinoma cells [35].

The mechanism of cell death induced by FK866 in EwS cells was not in the scope of this study and requires further investigations. Several different cell type specific mechanisms have previously been reported, including autophagy, oncosis, necrosis and apoptosis [25, 28, 31, 40]. Absence of PARP1 cleavage excludes classical apoptosis for FK866 induced loss of EwS viability. This conclusion is consistent with findings by Del Nagro *et al*. for cell types with rapid ATP depletion in response to NAMPT inhibition [25].

Cancer cells show increased metabolism through aerobic glycolysis and higher NAD turnover in order to sustain fast proliferation, relative genomic stability, persistent DNA repair [2, 3], and higher activity of the NAD-dependent deacetylase SIRT1 [49]. Elevated SIRT1 expression was found in hepatocarcinoma cells when compared to non-cancerous hepatocytes [49], and NAMPT inhibition led to cell death via the AMPK/mTOR pathway, offering a novel strategy to prevent cancer cell growth *in vivo* [35]. Cancers expressing high amounts of NAD-dependent enzymes might therefore be ideal candidates for therapeutic targeting of tumor cell NAD metabolism. We have previously shown that high SIRT1 positivity in EwS is associated primarily with metastases and is also present in primary EwS tumors and cell lines [16]. In addition, several reports revealed the importance of PARP1 for EwS tumor development and progression [15, 50]. High abundance of SIRT1 and PARP1, two major mammalian NAD consumers [51], may be linked to the striking sensitivity of EwS cells to NAMPT inhibition compared to Non-EwS cell lines (Figure 4B). However, NAMPT protein expression did not allow predicting the susceptibility of EwS cells to NAMPT inhibitors, as it remained unaffected by EWS-FLI1 protein levels. This is in concordance with other studies in CLL [31] and multiple myeloma cell lines [30] where NAMPT protein levels and cytotoxic response to FK866 did not correlate. Though NAMPT activity tended to increase in EFL cells, the lack of significance suggested that elevated NAD levels in absence of EWS-FLI1 cannot be fully explained by increased enzymatic activity of NAMPT. This finding may at least partially be explained by EWS-FLI1 knockdown-induced growth inhibition [36, 52]. We also found decreased basal respiration and diminished maximal respiratory capacity in EFL cells consistent with reduced cellular metabolism when EWS-FLI1 levels were low. As discussed recently at the 2^nd^ European Ewing Sarcoma Research Summit [53], stochastic variations in EWS-FLI1 expression may translate into differential metastatic behavior, since EFL cells were demonstrated to have drastically increased migratory and metastatic potential [54]. Whether variations in metabolic activity as described here for EWS-FLI1 knockdown may contribute to EwS progression remains to be investigated.

With regard to the mechanism of NAMPT inhibitor-induced vulnerability of EwS cells, we clearly identified the importance of NAD salvage pathways by demonstrating extensive NAD and ATP depletion, accompanied by loss of mitochondrial integrity and glycolytic impairment. All of these described consequences of FK866 treatment were rescued by addition of NA (or NMN) suggesting that salvage regeneration of NAD is central for energy homeostasis in EwS cells. Consequently, it is not surprising that tumor cells which rely on rapid NAD turnover and glycolysis are hit exceptionally hard by exhaustion of NAD [55]. This was confirmed for several hematologic and solid tumor models, where NAMPT inhibitors FK866 and CHS828 showed potent anticancer activity [10, 25, 26, 32, 35]. Despite these promising *in vitro* results, clinical phase I/II trials with these compounds as mono-therapy failed to exert significant anticancer activity in patients with solid tumors, among them renal and ovarian carcinoma, soft tissue sarcoma, prostate cancer, hepatocarcinoma, melanoma, and colorectal cancer [56–58]. In these studies, various side effects including thrombocytopenia, lymphopenia, anaemia, and various gastrointestinal symptoms limited dose escalation and, thus, contributed to disappointing trial outcomes [44]. In addition, low bioavailability for FK866 *in vivo* and rapid intravenous clearance prohibited sufficient drug delivery [56], which also highlights the need for second generation NAMPT inhibitors with increased efficacy, and some promising candidates are already on the horizon [59]. However, the exquisite sensitivity of EwS cells to low nanomolar concentrations of FK866 may alleviate bioavailability and toxicity problems of NAMPT inhibitors. FK866 concentrations effective against EwS cells *in vitro* are in the range of serum concentrations achieved for CHS828 below the maximum tolerated doses (1.5-10.7 nM) for a single oral administration of 150 mg and 500 mg, respectively [56–58]. In addition, combination chemotherapy with agents potentiating cancer cell specific NAD depletion is currently discussed [44, 60]. Intriguingly, one approach combines NAMPT inhibition with PARP-activating chemotherapeutics in order to boost the effect of intracellular NAD depletion [60, 61]. Eligible compounds for this approach comprise (a) temozolomide, a prototypic DNA damaging agent, already tested in combination with PARP inhibition in EwS cells [62, 63], (b) other DNA-damaging agents, such as MNNG [61] or β-lapachone [64], that induce PARP activation, or (c) drugs that have an intrinsic NAD-depleting effect [61].

In conclusion, we propose the use of low dose NAMPT inhibitors in combination chemotherapy of EwS due to its exquisite EWS-FLI1 dependent sensitivity to NAD depletion. Synthetic lethality of NAMPT inhibition with the presence of this oncogene may allow for an innovative new treatment option worthy of further exploration.

## MATERIALS AND METHODS

### Materials

Cell culture media (RPMI-1640, DMEM), fetal calf serum (FCS), and penicillin-streptomycin were purchased from Gibco, Life Technologies (Carlsbad, CA, USA). Blasticidin and zeocin were from InvivoGen (San Diego, CA, USA), doxycycline and fibronectin from Sigma Aldrich (Darmstadt, Germany). FK866 was obtained from AdipoGen (San Diego, CA, USA), nicotinic acid (NA), nicotinamide mononucleotide (NMN), and camptothecin were purchased from Sigma-Aldrich (Darmstadt, Germany). Mitochondrial stress test and glycolysis stress equipment and test reagents were obtained from Seahorse Biosciences (North Billerica, MA, USA).

### Cell culture

EwS cell lines STA-ET-2.2 and STA-ET-11 (provided by P.F. Ambros), RM-82 (provided by F. Van Valen), TC32 and TC252 (provided by T. Triche), SK-N-MC, A673, and a stably transfected subclone of A673 cells with a doxycycline-inducible shRNA against the EWS-FLI1 fusion protein (further referred to as A673sh) were used [65]. Other cell lines such as PC-3 (prostate cancer), HEK293 (human embryonic kidney), HeLa (cervix carcinoma), CLB-MA (neuroblastoma), U2OS (osteosarcoma), and MSCs were used for viability assays. All cells were grown at 37°C in a humidified atmosphere of 95% air and 5% CO_2_. FK866 was dissolved in DMSO and used at concentrations and time periods as indicated in the figures. FK866 was either used alone or in combination with NA (25 μM) and NMN (500 μM). NA and NMN were dissolved in PBS and added to the medium 6 h before the addition and throughout treatment with FK866.

### DNA-synthesis and viability assays

Cell viability was analyzed using the colorimetric thiazolyl blue tetrazolium bromide (MTT) metabolic activity assay. In brief, cells (0.4-1.0 × 10^4^ cells per well) were cultured in 96-well plates and exposed to different concentrations of FK866. DMSO (solvent)-treated cells served as negative control group. For the inducible EWS-FLI1 knockdown via shRNA, doxycycline treatment (1μg/mL) was started 24 h prior to drug treatment and the cells were kept in doxycycline until processing. 72 h after compound treatment, 20 μL of MTT solution (Sigma-Aldrich, Darmstadt, Germany; 5 mg/mL in PBS) were added and plates were incubated for 3 h at 37°C. Half maximal inhibitory concentration (IC_50_) values were determined by fitting a dose response curve to the data points using non-linear regression analysis (variable slope; four parameters) using GraphPad Prism 5.02 (Windows; GraphPad Software Inc.) software. Experiments were performed at least three times in triplicates.

### Soft agar assays

Cells were seeded in triplicates at 3 × 10^4^ cells per well in a 6-well plate. After re-suspension in 0.3% agar in 10% FCS, and in medium with or without 2 nM FK866, cells were plated in 0.6% agar-coated dishes. A top layer containing 0.6% agar was then added. Cells were fed every three days by placing three drops of medium (no inhibitor) on the top layer. Colony formation was examined after 21 days in three soft agar experiments.

### Cell death analysis

Adherent and floating cells were analyzed after 24, 48, and/or 72 h of 5 nM inhibitor treatment with the AnnexinV Apoptosis Detection Kit APC (eBioscience, San Diego, CA, USA). AnnexinV and DAPI (Sigma-Aldrich, Darmstadt, Germany) staining was performed according to manufacturer's instructions and FACS Fortessa and the FACS Diva™ software (BD Biosciences, San Jose, CA, USA) were used for quantification purposes. As positive control, apoptosis was induced via camptothecin (1 μM) for 24 or 48 h.

### NAD measurement

NAD and NADH levels were determined individually using the NAD/NADH-Glo Assay™ (Promega, Fitchburg, WI, USA) according to manufacturer's instructions. NAD serial standard dilutions were made from the concentrated NAD^+^ stock (Sigma-Aldrich, Darmstadt, Germany). In parallel, total protein was measured with the bicinchoninic assay (BCA) assay kit (Thermo Fisher Scientific, Waltham, MA, USA) and NAD levels were normalized to μg protein.

### ATP measurement

ATP levels were measured with the luminescent-based CellTiter-Glo® Luminescent Cell Viability Assay (Promega, Fitchburg, WI, USA) according to manufacturer's instructions. Serial ATP standard solutions were performed for each individual assay with ATP disodium salt from Sigma-Aldrich (Darmstadt, Germany). In parallel, total protein was measured with the BCA assay kit (Thermo Fisher Scientific, Waltham, MA, USA) and ATP levels were normalized to μg protein.

### NAMPT activity measurement

NAMPT activity was measured by the conversion of ^14^C-labeled NAM to ^14^C-NMN as previously described [35]. In brief, pelleted cells were re-suspended in 0.1 M sodium phosphate buffer, pH 7.4, and broken up by ultrasound on ice. 10 μg of lysate was used for the subsequent procedure and radioactivity of ^14^C-NMN was quantified in a liquid scintillation counter in disintegrations per minute (dpm) (Wallac 1409 DSA, PerkinElmer, Waltham, MA, USA). NAMPT activity was normalized to total protein concentrations measured by BCA protein assay (Thermo Fisher Scientific, Waltham, MA, USA).

### Immunoblot analysis

Total proteins were resolved by 6.5% or 8.5% SDS-polyacrylamide gel electrophoresis and processed for immunoblotting according to standard protocols. Antibodies used were FLI1 from MyBiosource (#300723, San Diego, CA, USA), GAPDH by Ambion (#4300, Thermo Fisher Scientifc, Waltham, MA, USA), NAMPT from Santa Cruz Biotechnology (#130058, Santa Cruz, CA, USA), and Vinculin from Sigma (#V4505, Darmstadt, Germany). Blot detection was performed with the LI-COR Odyssey Infrared Imaging System (LI-COR Biosciences, Lincoln, NE, USA).

### Mitochondrial respiration measurements (oxygen consumption rate - OCR)

Mitochondrial respiration was assessed using a Seahorse XF24 Extracellular Flux Analyzer (Seahorse Biosciences, North Billerica, MA, USA) according to the manufacturer's protocol. In brief, 1×10^4^ A673sh cells or 9×10^3^ TC32 cells were seeded per well in a 24-well XF24 microplate from Seahorse Biosciences. Two wells were left cell free for background control measurements. Cells were grown for 48 to 72 hours and subjected to flux analysis. For this purpose, cells were equilibrated with DMEM lacking bicarbonate and HEPES at 37°C for one hour in an incubator without CO_2_ prior to measurement. Basal oxygen consumption rate (OCR) was determined followed by sequential treatments with oligomycin (1 μM), carbonyl cyanide 4-trifluoromethoxyphenylhydrazone (FCCP, 2 μM), and antimycin (1 μM) + rotenone (1 μM). A minimum of three wells was used per condition in any given experiment and measurements were normalized to protein content for each well. For protein quantification with BCA assay (Thermo Fisher Scientific, Waltham, MA, USA) cells were lysed in 2% SDS for 15 min at 37°C post flux analysis and before adding the BCA reagent. Absorbance was measured at 595 nm, and oxygen consumption rate per well was determined as pmol/min/μg protein.

### Glycolytic stress analysis (extracellular acidification rate - ECAR)

Rate of glycolysis was determined according to the Seahorse Bioscience protocol (Seahorse Biosciences, North Billerica, MA, USA). In brief, on the day of the experiment cells were incubated with bicarbonate-free low-buffered glycolysis assay medium (glucose-free, GlutaMAX 2 mM, pH 7.4) for one hour at 37°C in the absence of CO_2_ prior to the beginning of the assay. For the glycolytic analysis, the following sequential injections of drugs were used: Glucose (15 mg/mL), oligomycin (1 μg/mL) and 2-deoxy-D-glucose (2-DG, 1 M). Glycolysis was determined as extracellular acidification rate (ECAR) in mpH/min/μg protein. Protein quantification was performed with the BCA assay kit as described for mitochondrial respiration measurements above.

### Statistical analysis

Results are shown as representative images or as means ±SD of at least three independent experiments. Data were analyzed using the unpaired t-test with Welch's correction or with the one-sample t-test using the Prism 5 for Windows (version 5.02) statistical software (GraphPad Prism Software Inc.). Data shown in graphical format represent the means (±SD), and a *P*-value of 0.05 was considered statistically significant.

## SUPPLEMENTARY MATERIALS FIGURES AND TABLES



## Abbreviations

2-DG: 2-deoxy-D-glucose
ADP: adenosine diphosphate
AnnexinV^+^: AnnexinV positive
ATP: adenosine triphosphate
BCA: bicinchoninic acid
BR: basal respiration
DMSO: dimethyl sulfoxide
DNA: deoxyribonucleic acid
ECAR: extracellular acidification rate
EFH: EWS-FLI1-high
EFL: EWS-FLI1-low
EwS: Ewing sarcoma
FCCP: carbonyl cyanide 4-trifluoromethoxyphenylhydrazone
FCS: fetal calf serum
GC: glycolytic capacity
GR: glycolytic rate
IC_50_: half-maximal inhibitory concentration
MRC: maximal respiratory capacity
MSC: mesenchymal stem cell
NA: nicotinic acid
NAD: nicotinamide adenine dinucleotide
NAM: nicotinamide
NAMPT: nicotinamide phosphoribosyltransferase
NaMN: nicotinic acid mononucleotide
NMN: nicotinamide mononucleotide
NR: nicotinamide riboside
OCR: oxygen consumption rate
PARP: poly-(ADP-ribose) polymerase
PARPi: PARP inhibitor
PRPP: 5-phosphoribosyl-1-pyrophosphate
SD: standard deviation
SDS: sodium dodecyl sulfate
