# Supplementary material for: EWS-FLI1 confers exquisite sensitivity to NAMPT inhibition in Ewing sarcoma cells
Source: Oncotarget. 2017 Feb 1;8(15):24679–93. doi: 10.18632/oncotarget.14976 (PMC5421879; doi:10.18632/oncotarget.14976)
Supplement: Supplementary file 1 [file oncotarget-08-24679-s001.pdf]

EWS-FLI1 confers exquisite sensitivity to NAMPT inhibition in Ewing sarcoma cells

SUPPLEMENTARY FIGURES AND TABLE

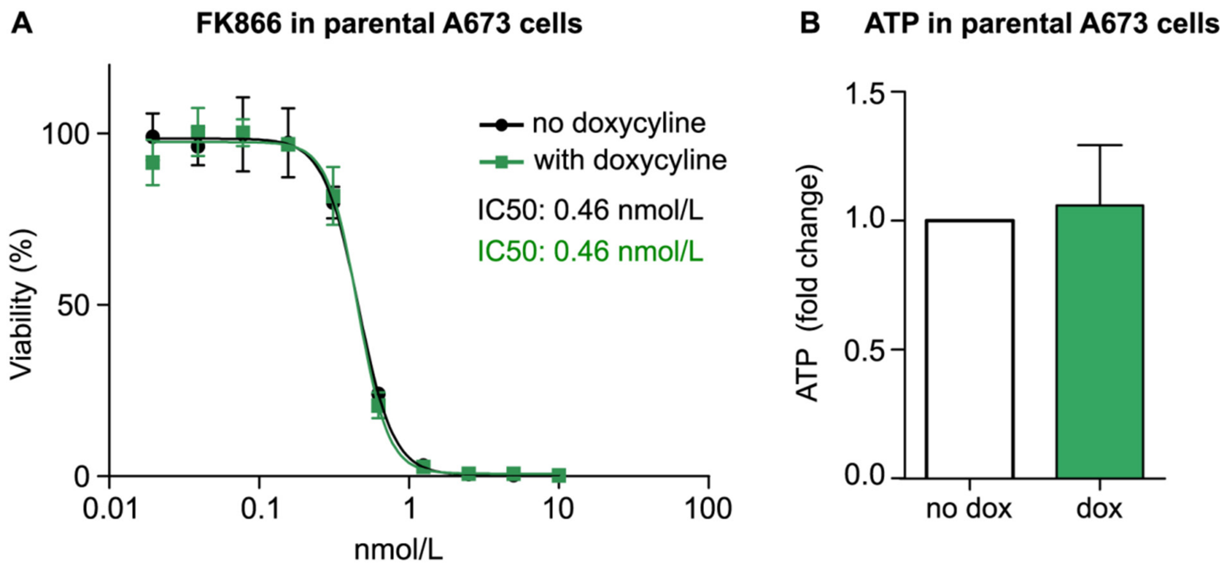

Supplementary Figure 1: A. Viability assay for FK866 treatment in parental A673 cells. 4×10^3 A673 cells were seeded in each well of 96-well plates, treated with doxycycline the next day for 24 hours, and subsequently compound treated for additional 72 h. **B.** ATP measurement in parental A673 cells. 4×10^3 A673 cells were seeded in 96-well plates and were treated (dox) or not (no dox) for 96 hours. Results were normalized to total protein amount and are displayed as fold change over control (no dox). Half maximal inhibitory concentration (IC_{50}) values were determined by fitting a dose response curve to the data points using non-linear regression analysis (variable slope; four parameters) using GraphPad Prism 5.02 (Windows; GraphPad Software Inc.) software. Data represent the means ± SD from 4 technical replicates.

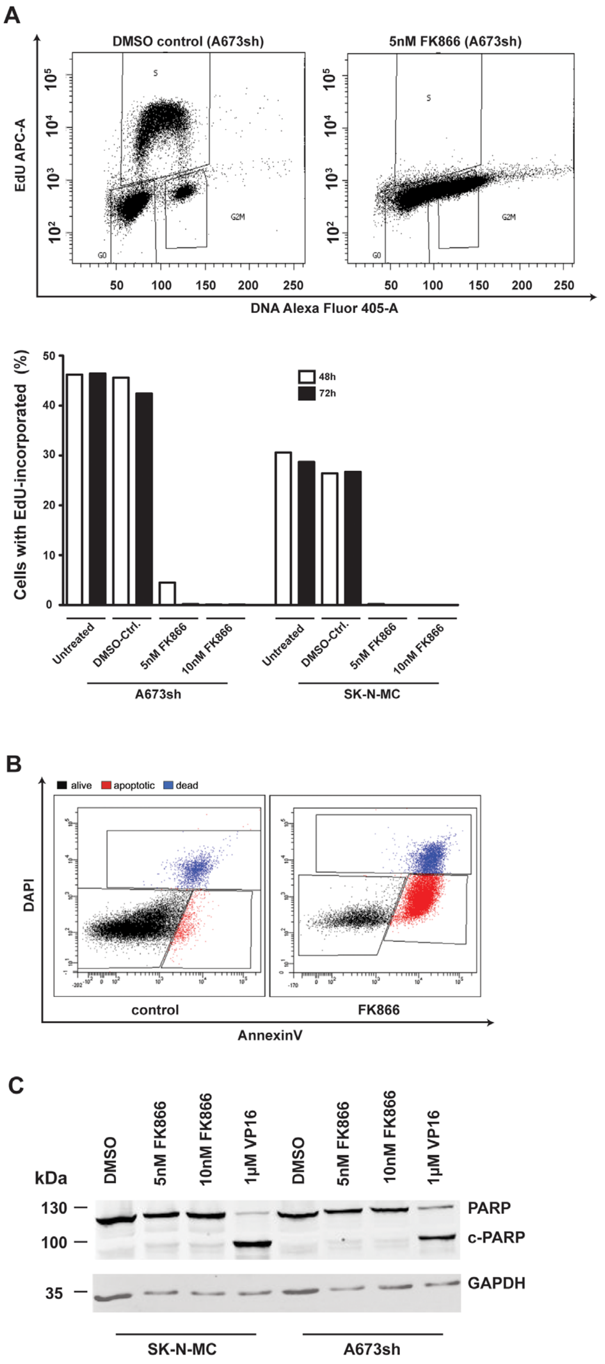

Supplementary Figure 2: A. Evaluation of DNA synthesis upon FK866 treatment (5 nM; 72 h) by EdU incorporation assay. Scatter plots show results from one representative experiment (upper panel) and the percentages of cells with EdU incorporation in S-phase of the cell cycle (lower panel) of three independent experiments. The Click-iT EdU Alexa Fluor 647 Flow Cytometry Assay kit (Invitrogen, Molecular Probes, Life Technologies, Vienna, Austria) was used following the manufacturer's instructions to measure EdU incorporation. Briefly, about 650,000 cells (FK866-treated and DMSO controls) were fixed and permeabilized with reagents supplied in the kit. Stainings for DNA (Cell cycle 405 blue) and EdU detection (Alexa-Fluor 647) were carried out as recommended by the manufacturer and analysed by flow cytometry. **B.** Flow-cytometric determination of cell death in TC32 cells upon FK866 treatment (5 nM, 72 h) by combined AnnexinV/DAPI staining. The presence of a AnnexinV-positive/DAPI-negative cell population is indicative of apoptosis-like cell death with maintenance of cell membrane integrity at early phases. **C.** Absence of PARP1 cleavage in response to FK866 treatment (5 and 10 nM for 72 h) suggests lack of caspase activation during NAMPT inhibition induced cell death. Etoposide (VP16) treatment is shown for positive control.

Supplementary Table 1: Gene fusion and p53 status of EwS and non-EwS cell lines

EwS cells	fusion type	p53 status
A673sh / A673	*EWS-FLI1* (exon 7/6)	mt p53
TC32	*EWS-FLI1* (exon 7/6)	wt p53
STA-ET-2.2	*EWS-FLI1* (exon 9/4)	mt p53
SK-N-MC	*EWS-FLI1* (exon 7/6)	mt p53
TC252	*EWS-FLI1* (exon 7/6)	wt p53
STA-ET-11	*EWS-ERG*	wt p53
RM-82	*EWS-ERG*	mt p53
**Non-EwS cells**		
PC-3	*TMPRSS2-ERG*	mt p53
HEK293	-	mt p53
HeLa	-	mt p53
CLB-MA	-	wt p53
U2OS	-	wt p53
MSC	-	wt p53
